# Endothelial AGO1 deficiency reduces breast cancer burden in mice

**DOI:** 10.1007/s10456-026-10048-6

**Published:** 2026-06-12

**Authors:** Xuejing Liu, Alonso Tapia, Dongqiang Yuan, Xiaofang Tang, Yingjun Luo, Naseeb Kaur Malhi, Muxi Chen, Kwan Ho Law, Mohamad Haidar, Skylar Giacobetti, Jiawei Sun, Anthony Park, Saul J. Priceman, Zhen Bouman Chen

**Affiliations:** 1 Department of Diabetes Complications and Metabolism, Beckman Research Institute, Arthur Riggs Diabetes and Metabolism Research Institute, City of Hope, Duarte, CA USA; 2 Irell and Manella Graduate School of Biological Sciences, City of Hope, Duarte, CA USA; 3 Department of Hematology and Hematopoietic Cell Transplantation, City of Hope, Duarte, CA USA; 4 Department of Medicine, KSOM/NCCC Center for Cancer Cellular Immunotherapy Research, Keck School of Medicine of USC, Los Angeles, CA USA

**Keywords:** Argonaute 1 (AGO1), Endothelial cells (ECs), Tumor immunology, Breast cancer

## Abstract

Endothelial cells (ECs) are crucial in cancer development and progression, partly by regulating tumor angiogenesis and immune modulation. As a key component of the RNA-induced silencing complex, Argonaute 1 (AGO1) regulates tumor biology, yet the specific function of AGO1 within ECs in the tumor microenvironment remains undefined. Here, we investigated the effects of endothelial-specific AGO1 knockout (EC-AGO1-KO) on tumor vascularization and immune regulation in a mouse syngeneic breast cancer model induced by E0771 cells. EC-AGO1-KO mice exhibited significantly reduced tumor burden compared to their wild-type (WT) littermates, accompanied by reduced vascularization and enhanced immune cell infiltration. Histological and single-cell RNA sequencing analyses revealed increased infiltration of CD8⁺ T cells and macrophages in EC-AGO1-KO tumors, indicative of an immunostimulatory microenvironment. In vitro, AGO1 knockdown in mouse ECs co-cultured with E0771 tumor cells led to higher levels of *Cxcl10* and *Vcam1* expression, suggesting a pro-inflammatory and leukocyte-recruiting effect. Together, these findings identify endothelial AGO1 as a key regulator of tumor vasculature and immune homeostasis in breast cancer, suggesting that targeting endothelial AGO1 may represent a novel therapeutic strategy to modulate tumor vasculature while enhancing anti-tumor immunity.

**Supplementary Information:**

The online version contains supplementary material available at 10.1007/s10456-026-10048-6.

## Dear Editor

Endothelial cells (ECs) are critical for tumor development and metastasis. Argonaute 1 (AGO1), a key component of the RNA-induced silencing complex, is integral to gene regulation [1]. Several studies have explored AGO1 function in tumor cells and demonstrated its context-dependent effects [2]. We have previously shown that suppression of AGO1 in non-tumor ECs enhances VEGF-A expression and angiogenesis under hypoxia [3], and that EC-conditional AGO1 knockout (EC-AGO1-KO) mice are protected from diet-induced obesity, hyperlipidemia, and atherosclerosis [4, 5]. However, the role of endothelial AGO1 in tumor vasculature and immune modulation remains unclear.

**Fig. 1:**
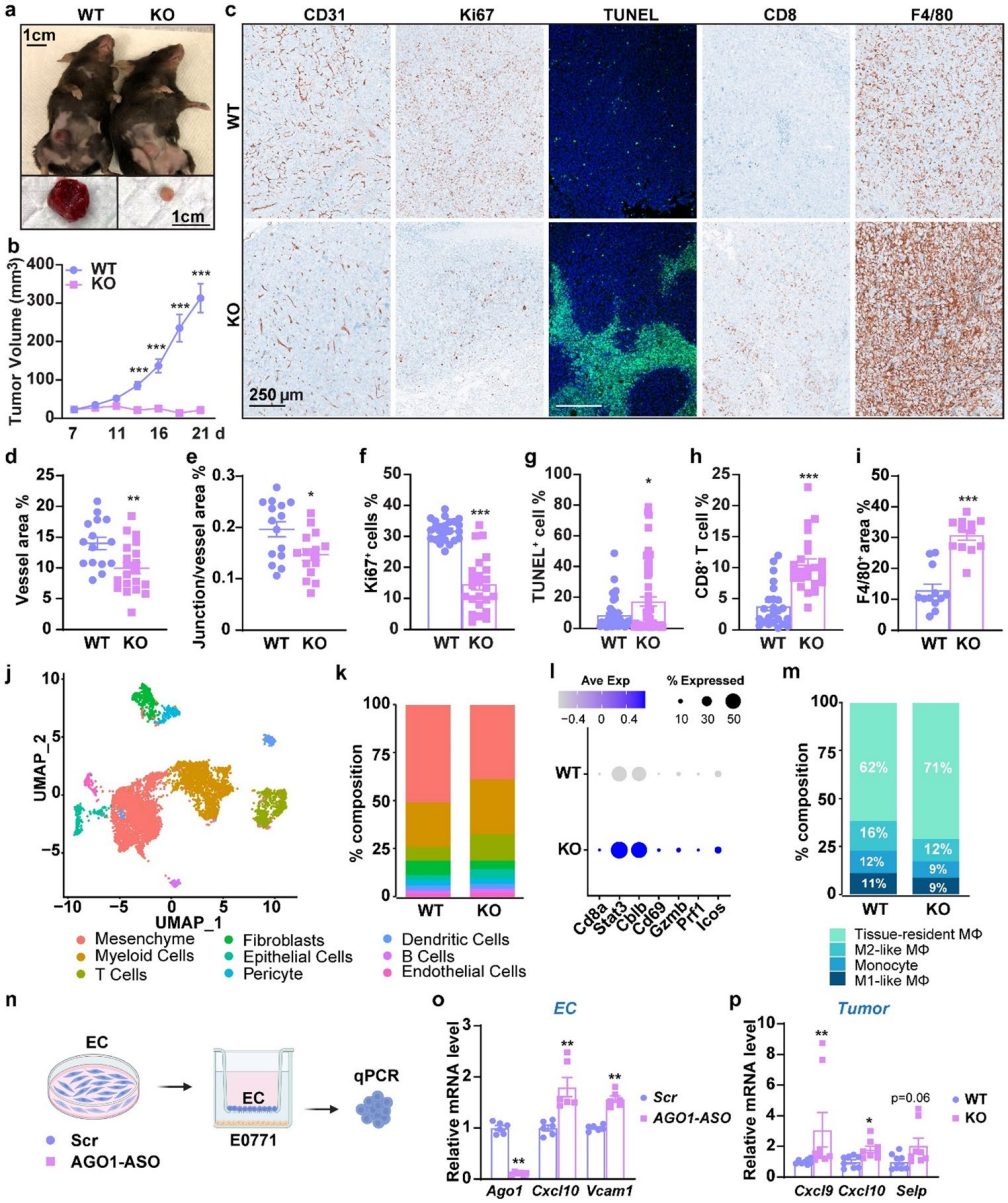
EC-AGO1 inhibition impairs tumor angiogenesis, modulates the immune microenvironment, and inhibits tumor growth in vitro and in vivo.** a**–**m** Female EC-AGO1-KO mice and their WT littermates (16-week-old) were injected with E0771 breast cancer cells. **a** Representative image of mice with mammary tumors (upper panel) and the dissected tumors (lower panel) at day 21. **b** Quantification of tumor size over time (*n* = 10/group). **c** Representative images of CD31, Ki67, TUNEL, CD8, and F4/80 staining of tumor harvested at day 21.** d**–**e** Quantification of vessel percentage and junction/vessel area (24 views from 3–4 tumors/group).** f**–**i** Quantification of Ki67, TUNEL, and CD8 (24–56 views from 3–4 tumors/group) and F4/80-positive area (12 views from 3–4 tumors/group).** j**–**m** scRNA-seq of tumors (*n* = 5–7 tumors per group). **j** Uniform manifold approximation and projection (UMAP) of scRNA-seq data, classified by major non-tumor cell types. **k** Cell composition (%) in the tumor scRNA-seq data separated by WT vs. KO mice. **l** Dot plot showing expression of activation marker genes in T cell populations in the scRNA-seq. **m** Cell composition (%) of macrophage (MΦ) subtypes in tumors from the WT and KO mice. **n** Schematic showing mouse ECs (MS1) transfected with control scramble (Scr) or AGO1-antisense oligos (ASO) (20 nM) for 48 h, followed by 24 h co-culture with E0771 cells in Transwell. **o**,** p** mRNA levels of marker genes were quantified by qPCR in ECs (**o**) and tumors (**p**). Data are presented as mean ± SEM in (**b**,** d**–**i**,** o**,** p**). *p < 0.05, **p < 0.01, ***p < 0.001 between the indicated groups based on Student’s t-test (**b**,** d**–**i**,** o**,** p**)

To address this, we implanted syngeneic E0771 breast cancer cells into the mammary fat pad of WT and EC-AGO1-KO mice. In the KO mice, AGO1 deletion was restricted to ECs and absent in monocytes [4, 5], minimizing potential confounding contribution from hematopoietic cells differences to the observed phenotypes. By Day 7, all mice developed palpable tumors (> 10 mm^3^). However, by Day 21, EC-AGO1-KO mice exhibited significantly reduced tumor volumes compared to WT controls, with tumors appearing paler and undetectable in approximately 50% of the mice (Fig. 1a, b; Fig. S1; Fig. S2). Circulating estradiol levels were comparable between the WT and KO mice, suggesting the observed phenotype was not attributable to hormonal variation (Fig. S3). Immunohistochemical (IHC) analysis revealed a significant reduction in vascularization in EC-AGO1-KO tumors, evidenced by CD31 staining and the quantification of vessel area and junction density (Fig. 1c-e). Concordantly, EC-AGO1-KO tumors exhibited reduced Ki67 staining (Fig. 1c, f) and increased TUNEL staining compared to WT controls (Fig. 1c, g), suggesting decreased tumor cell proliferation and enhanced apoptosis. These differences were observed as early as Day 14 (when all mice carried tumors, Fig. S4). The increased tumor cell death in the KO mice prompted us to examine immune cell infiltration, revealing elevated CD8^+^ T cells and F4/80^+^ macrophage infiltration compared to WT controls (Fig. 1c, h, i).

Single-cell RNA sequencing (scRNA-seq) of tumor samples revealed 9 non-tumor cell clusters, including the immune cells (Fig. 1j; Fig. S5a), with EC-AGO1-KO tumors showing a marked increase in T cells and macrophages (Fig. 1k; Table S1). Consistent with the IHC data, activated T cell marker genes (*Cd8a*,* Stat3*,* Cblb*,* Gzmb)* were increased in KO tumors (Fig. 1l). Within the macrophage population, tissue-resident macrophages (marked by *Cadm1*,* Mertk*,* Zeb2*,* and Cd53*) were increased and immunosuppressive M2-like macrophages (marked by *Mrc1 and Arg1*) were reduced (Fig. 1m; Fig. S5b). Furthermore, macrophages in KO tumors showed higher expression of immunostimulatory *Cxcl9/10* and weak expression of immunosuppressive *Arg1* and *Il10* (Fig. 1m; Fig. S5c–f). These results suggest a more immunostimulatory microenvironment in the EC-AGO1-KO tumor.

Given the limited number of ECs captured in the scRNA-seq dataset, we employed an EC-cancer cell co-culture model to define EC-intrinsic gene programs. Mouse ECs with AGO1-knockdown (AGO1-KD) co-cultured with E0771 cells exhibited increased *Cxcl10* and *Vcam1* expression compared to scramble-treated control ECs (Fig. 1n, o). Supporting these in vitro findings, tumors from EC-AGO1-KO mice expressed higher levels of *Cxcl9*, *Cxcl10*, and *Selp* (encoding P-selectin*)* expression (Fig. 1p), collectively suggesting that AGO1 suppression in ECs promotes leukocyte recruitment within the tumor microenvironment. In contrast, expression of *Kdr* and *Nos3* remained unchanged in AGO1-KD ECs either cultured alone or co-cultured with E0771 cells (Fig. S6), suggesting that the reduced tumor burden in EC-AGO1-KO mice is unlikely to be explained by vascular dysfunction.

To investigate potential paracrine effects of AGO1-KD ECs on tumor cells, we treated E0771 cells with conditioned media (CM) from ECs. Compared to the scramble control, CM from ECs with AGO1-KD reduced the E0771 cancer cell migration, with minimal impact on their proliferation (Fig. S7a–e). At the molecular level, AGO1-KD in ECs decreased expression of *CXCR4*, a metastasis-associated chemokine receptor that also plays a role in primary tumor expansion, and increased *RUNX1T1*, a tumor-suppressive transcriptional repressor, in E0771 cells (Fig. S7f–i). These data suggest that EC-AGO1 may also regulate cancer development through a direct effect on the tumor cells, which warrants future investigation.

Collectively, our findings reveal a previously unrecognized role of endothelial AGO1 in tumor progression. EC-AGO1 inhibition decreases tumor vascularization, increases immune cell infiltration and reduces tumor burden in a mouse breast cancer model. These results highlight the pivotal role of ECs in shaping tumor behavior and microenvironment and underscore AGO1 as a regulatory node in the EC-tumor axis. Further studies are needed to establish its generalizability across tumor types. Targeting EC-AGO1 may offer a novel therapeutic avenue to disrupt aberrant tumor angiogenesis, promote immune cell infiltration, and enhance the efficacy of existing cancer therapies.

## Supplementary Information

Below is the link to the electronic supplementary material.

Supplementary Material 1

## Data Availability

The scRNA-seq data generated in this study are available at GEO with accession number GSE310768.
