# Supplementary material for: Endothelial AGO1 deficiency reduces breast cancer burden in mice
Source: Angiogenesis. 2026 Jun 12;29(3):43. doi: 10.1007/s10456-026-10048-6 (PMC13263250; doi:10.1007/s10456-026-10048-6)
Supplement: Supplementary file 1 — Supplementary Material 1 [file 10456_2026_10048_MOESM1_ESM.docx]

**SUPPLEMENTARY INFORMATION**

**Endothelial AGO1 Deficiency Reduces Breast Cancer Burden in Mice**

Xuejing Liu^1*^, Alonso Tapia^1,2*^, Dongqiang Yuan^1^, Xiaofang Tang^1^, Yingjun Luo^1^, Naseeb Kaur Malhi^1^, Muxi Chen^1,2^, Kwan Ho Law^1^, Mohamad Haidar^1^, Skylar Giacobetti^1^, Jiawei Sun^1^, Anthony Park^3^, Saul J. Priceman^3,4^, Zhen Bouman Chen^1,2^

^1^Department of Diabetes Complications and Metabolism, Arthur Riggs Diabetes and Metabolism Research Institute, Beckman Research Institute, City of Hope, Duarte, CA, USA.

^2^Irell and Manella Graduate School of Biological Sciences, City of Hope, Duarte, CA, USA.

^3^Department of Hematology and Hematopoietic Cell Transplantation, City of Hope, Duarte, CA, USA

^4^Department of Medicine, KSOM/NCCC Center for Cancer Cellular Immunotherapy Research, Keck School of Medicine of USC, Los Angeles, CA, USA

^*^These authors contributed equally

Correspondence: zhenchen@coh.org (Z.B.C.)

**MATERIALS AND METHODS**

**Study Approval**

All animal experiments conducted were approved by the Institutional Animal Care and Use Committees at City of Hope (#17010) and Institutional Biosafety Committee (#16023).

**Mouse Models**

EC-AGO1-KO and their WT littermates were generated by crossbreeding VE-Cadherin-Cre (B6.FVB-Tg [Cdh5-cre]7 Mlia/J) and AGO1^flox/flox^ (Ago1tm1.1Tara/J) mice, both with C57BL/6 background at City of Hope as described [1]. At end points of each experiment, mice were euthanized with CO_2_ inhalation.

**E0771 breast cancer** **syngeneic model**

The WT and KO littermates from the same breeders were housed in the same cages and E0771 breast cancer cells (per mouse 1 × 10^6^ cells in 100 µL PBS/Matrigel (Sigma, CLS356231) mixed at a 1:1 ratio) were injected in a blinded fashion into the fourth mammary fat pad of 16-week-old female mice. The tumor sizes were measured one week after injection using a digital caliper and the volumes were calculated using the formula Tumor volume = length×width×height×1/2. The mice were humanely euthanized if the tumor volume reached 1500 mm^3^ within 21 days or were anesthetized 21 days after tumor induction (unless indicated specifically). Total 30 mice per group were analyzed (**Fig. S2** showing the tumor volume at day 21), 10 mice per group were randomly selected for the tumor weight measurements, and the rest used for histological analysis and scRNA-seq.

**Histology and immunohistochemistry (IHC) analysis**

Tumors (>30 mg, >30 mm³) were harvested and fixed in 10 % neutral buffered formalin. Dehydration, clearing, and paraffinization were performed on a Tissue-Tek VIP Vacuum Infiltration Processor (SAKURA). The samples were embedded in paraffin using a Tissue-Tek TEC Tissue Embedding Station (SAKURA) and sectioned and put on positively charged glass slides. The IHC stains were performed using a Ventana Discovery Ultra IHC automated stainer (Ventana Medical Systems, Roche Diagnostics, Indianapolis, USA). Briefly, the slides were deparaffinized, rehydrated, and incubated with endogenous peroxidase activity inhibitor and antigen retrieval solution. Anti-mouse CD31 (Cat#: AC-0083A, Eptomics), F4/80 (Cat#: 70076, Cell Signaling), anti-mouse CD8 rabbit monoclonal antibody (Cat#: 98941, Cell signaling), or anti-mouse Ki67 rabbit monoclonal antibody (Cat#: 790-4286, Ventana, RTU) were incubated, followed by DISCOVERY anti-rabbit HQ and DISCOVERY anti-HQ-HRP (Ventana). The antibodies were visualized by DISCOVERY ChromoMap DAB Kit (Ventana) and counterstained with hematoxylin and cover slipped. Whole slide images were acquired with a NanoZoomer S360 Digital Slide Scanner (Hamamatsu) and viewed by NDP.view image viewer software. For vasculature analysis, images from CD31 IHC staining were quantified using AngioTool in a blinded fashion. Vessel area % and junction/vessel area % were acquired from 3-5 images per mouse. The percentages of CD8⁺ and Ki67⁺ cells, as well as the F4/80⁺ area, were quantified using QuPath.

**Measurement of circulating estradiol levels**

Blood was collected from female WT and EC-AGO1-KO mice at 8 weeks of age (n = 5 per group) at two time points of the estrous cycle (Day 1 and Day 4). Plasma was obtained by centrifugation at 4000 rpm for 10 min at 4 °C, and 50 µL aliquots were used for measurement. Circulating estradiol concentrations were quantified using the Mouse Estradiol Rapid ELISA Kit (Thermo Fisher Scientific, Cat# EELR013) following the manufacturer’s instructions. Briefly, plasma samples and standards were added to pre-coated wells, incubated with enzyme conjugate, and washed to remove unbound material before adding substrate solution. The reaction was stopped with termination buffer and absorbance was measured at 450 nm. Estradiol concentrations were calculated from the standard curve. Output values from the two cycle time points were averaged for each animal.

**Cell culture, transfection, and treatment**

Cells were kept at 37 °C, ventilated with 5 % CO_2_ and 21 % O_2_. Validated mouse breast cancer E0771 cell lines were grown in Dulbecco’s modified DMEM medium (Cat#: 11965092, Gibco) supplemented with 10 % fetal bovine serum (FBS), 2.5 % HEPES, and 1× antibiotics (Cat#: 15140122penicillin–streptomycin, Gibco). HUVECs (passages 5–8) were cultured in complete M199 medium supplemented with 10 % FBS (Sigma, M2520) and 1× antibiotics (penicillin–streptomycin, Gibco, 15140122). Mile Sven 1 (MS1) cells (a mouse microvascular EC line, passages 4-6) (American Type Culture Collection) were cultured in DMEM medium. In some experiments, ECs were pre-transfected with scramble (Scr) or AGO1-ASO (20 nM) using Lipofectamine RNAiMax. The medium was changed to M199 or DMEM 6 h after transfection, 48 h later, changed to fresh medium, then conditioned media (CM) were collected 24 hours later, filtered through a 0.45 μm filter, and mixed with DMEM at a 1:1 ratio for treatment of E0771 cells. In the Transwell (Corning, CLS3470) co-culture experiment, the MS1 cells were cultured in the upper chamber and were pre-transfected with Scr or AGO1-ASO, 48 h later, co-cultured with E0771 cells which were pre-seeded in the lower chamber for another 24 h. The sequence of Scr-ASO is 5'- +A*+A*+C* A*C*G*T*C*T*A*T*A*+C*+G*+C -3', AGO1-ASO 5'- +C*+T*+T* G*T*G* T*A*A* G*G*A* A*+T*+G* +T -3'.

**Scratch assay**

E0771 cells were seeded on 12-well plates and cultured until 80 % confluence. Cell monolayers were wounded with a 10 μL pipette tip to generate a constant diameter strip across the center of the well. After scratching, the cells were washed with PBS to remove cell debris and incubated with DMEM, CM-Scr, or CM-AGO1-ASO. Images were taken at stated timepoints, and areas lacking cells were determined by measurement using ImageJ in a blinded fashion.

**RNA extraction, quantitative PCR, and RNA-seq**

Total RNA was extracted from cells using TRIzol reagent (Invitrogen, cat # 15596026). cDNAs were synthesized using PrimeScript™ RT Master Mix containing both Oligo-dT primer and random hexamer primers. qPCR was performed with Bio-Rad SYBR Green Supermix using the Bio-Rad CFX Connect Real Time system. The primers used in this study are summarized in **Table S2**.

Cell suspensions for single-cell RNA-seq were prepared following a published protocol [2]. 1 µg of total RNA from three biological replicates was used for library preparation with the KAPA mRNA HyperPrep Kit (Roche, cat# KK8581). mRNA was enriched using mRNA capture beads, and libraries were prepared following the manufacturer’s protocol (KR0960-v6.17). The quality of cDNA libraries was assessed using the Agilent 4200 TapeStation System. Libraries were sequenced using the NovaSeq platform (Illumina) with 150-nt paired-end sequencing, achieving a depth of 50~100 million read pairs.

**Western blotting**

E0771 cells were homogenized in RIPA buffer (50 mM Tris-HCl, pH 8.0, 150 mM NaCl, 5 mM EDTA, 1 mM DTT, 1% NP-40, 0.1% SDS) with protease and phosphatase inhibitor cocktail (ThermoFisher, cat# 78442). Proteins were separated by SDS-PAGE and transferred onto PVDF membranes. Membranes were processed according to the ECL western blotting protocol (ThermoFisher). Images were captured using the Amersham Imager 680 (GE Healthcare) or ChemiDoc MP imaging system (Bio-rad). The gray values of Western blot bands were quantified using ImageJ. Antibodies included mouse CXCR4 monoclonal antibody (1:2000; Proteintech, cat# 60042-1-Ig), rabbit RUNX1T1 polyclonal antibody (1:5000; Proteintech, cat# 15494-1-AP), rabbit GAPDH monoclonal antibody(1:2000; CST, cat# 2118S), anti-rabbit IgG, HRP-linked antibody (1:5000; CST, cat # 7074), anti-mouse IgG, HRP-linked antibody (1:5000; CST, cat # 7076).

**Single-cell RNA sequencing**

Single-cell RNA sequencing (scRNA-seq) was performed on dissociated tumor tissues from EC-AGO1-KO and wild-type (WT) mice. Tumors from a total of 5 WT and 7 KO mice were used and individually processed except for the KO tumors with sizes <50 mm³ that were pooled. Five samples per WT/KO group were subjected to scRNA-seq and the subsequent analysis. Single-cell suspensions were prepared via enzymatic digestion using a previously established protocol [2] and processed using the 10x Genomics Chromium Single Cell 3’ v3.1 system. Libraries were aligned to the mm10 genome using Cell Ranger v7.1.1 to generate raw gene expression matrices. Data preprocessing and downstream analysis were performed using Seurat v4.3.0. Cells with fewer than 200 genes, more than 7,000 genes, or >10 % mitochondrial gene content were excluded. Following quality control, gene expression values were normalized and variance-stabilized using SCTransform, regressing out mitochondrial gene percentage and RNA content as confounders. Tumor cells were annotated following published studies and based on expression of genes associated with oncogenic and tumor-intrinsic transcriptional programs (e.g., *Myc* and *Trp53*) [3], as well as markers linked to developmental (*Esx1*) and stromal (*Aebp1*) signatures [4,5]. Annotated tumor cells were subsequently excluded from downstream analyses to enable focused characterization of non-tumor cell populations. The remaining cells were integrated across batches using Seurat’s SCTransform-based workflow. Dimensionality reduction was performed using principal component analysis (PCA), and significant principal components were selected based on elbow plots and jackstraw analysis. Cell clustering was performed using the Louvain algorithm with resolution optimization, and two-dimensional embedding was performed via Uniform Manifold Approximation and Projection (UMAP). Clusters were annotated based on canonical lineage markers for mesenchyme (*Pvt1, Fmnl2, Vim, Cdh13),* myeloid (*C1qa, C1qb*), T cells (*Gzmb, Ctla4, Il2ra*), fibroblasts (*Col1a1, Col1a2*), epithelial cells (*Gata3, Fos, Krt8*), pericyte (*Rgs5, Acta2, Pdgfrb*), dendritic cells (*Rel, Relb, Fscn1, Ccr7),* B cells (*Cd79a, Cd74*), and endothelial (*Pecam1*, *Cdh5*, *Cd34, Vwf*) populations. Myeloid compartments were subset and reclustered for higher-resolution analysis. Subclusters were annotated into tissue-resident macrophages, monocyte-derived M1 and M2 macrophages, and inflammatory monocytes based on differentially expressed genes (DEGs) and literature-derived marker sets [6-8].

Differential expression analysis was performed using Seurat’s FindMarkers function (Wilcoxon rank-sum test), with p-values adjusted by the Benjamini-Hochberg method. Cluster composition and gene expression distributions were visualized using ggplot2-based bar plots, violin plots, and dot plots.

**Statistical Analysis**

All *in vitro* data represent at least three independent experiments. All *in vivo* data represent experiments performed with numbers of mice as specified in the figure legends. Statistical analyses for data other than high-throughput sequencing were performed using GraphPad Prism. Two-group comparisons were performed using 2-sided Student’s t-test, and multiple-group comparisons were performed using ANOVA followed by Tukey’s post-hoc test. P values less than 0.05 were considered statistically significant.

**References:**

1. Tang X, Miao Y, Luo Y, Sriram K, Qi Z, Lin FM, Gu Y, Lai CH, Hsu CY, Peterson KL, Van Keuren-Jensen K, Fueger PT, Yeo GW, Natarajan R, Zhong S, Chen ZB (2020) Suppression of Endothelial AGO1 Promotes Adipose Tissue Browning and Improves Metabolic Dysfunction. Circulation 142 (4):365-379. doi:10.1161/CIRCULATIONAHA.119.041231

2. Rodriguez de la Fuente L, Law AMK, Gallego-Ortega D, Valdes-Mora F (2021) Tumor dissociation of highly viable cell suspensions for single-cell omic analyses in mouse models of breast cancer. STAR Protoc 2 (4):100841. doi:10.1016/j.xpro.2021.100841

3. Hanahan D, Weinberg RA (2011) Hallmarks of cancer: the next generation. Cell 144 (5):646-674. doi:10.1016/j.cell.2011.02.013

4. Eckel KL, Tentler JJ, Cappetta GJ, Diamond SE, Gutierrez-Hartmann A (2003) The epithelial-specific ETS transcription factor ESX/ESE-1/Elf-3 modulates breast cancer-associated gene expression. DNA Cell Biol 22 (2):79-94. doi:10.1089/104454903321515896

5. Li J, Ruan Y, Zheng C, Pan Y, Lin B, Chen Q, Zheng Z (2023) AEBP1 Contributes to Breast Cancer Progression by Facilitating Cell Proliferation, Migration, Invasion, and Blocking Apoptosis. Discov Med 35 (174):45-56. doi:10.24976/Discov.Med.202335174.6

6. Wu SZ, Al-Eryani G, Roden DL, Junankar S, Harvey K, Andersson A, Thennavan A, Wang C, Torpy JR, Bartonicek N, Wang T, Larsson L, Kaczorowski D, Weisenfeld NI, Uytingco CR, Chew JG, Bent ZW, Chan CL, Gnanasambandapillai V, Dutertre CA, Gluch L, Hui MN, Beith J, Parker A, Robbins E, Segara D, Cooper C, Mak C, Chan B, Warrier S, Ginhoux F, Millar E, Powell JE, Williams SR, Liu XS, O'Toole S, Lim E, Lundeberg J, Perou CM, Swarbrick A (2021) A single-cell and spatially resolved atlas of human breast cancers. Nat Genet 53 (9):1334-1347. doi:10.1038/s41588-021-00911-1

7. Xu L, Saunders K, Huang SP, Knutsdottir H, Martinez-Algarin K, Terrazas I, Chen K, McArthur HM, Maues J, Hodgdon C, Reddy SM, Roussos Torres ET, Xu L, Chan IS (2024) A comprehensive single-cell breast tumor atlas defines epithelial and immune heterogeneity and interactions predicting anti-PD-1 therapy response. Cell Rep Med 5 (5):101511. doi:10.1016/j.xcrm.2024.101511

8. Liu P, Ma W, Wang T, Lu J, Wang W, Wang Y, Tang Q, Di J, Bischof E, Zhao Q, Yu Z (2025) Single-cell RNA sequencing reveals the effects of mental stress on mouse mammary tumors and the tumor microenvironment. Cell Death Discov 11 (1):328. doi:10.1038/s41420-025-02619-1

**SUPPLEMENTAL FIGURES**


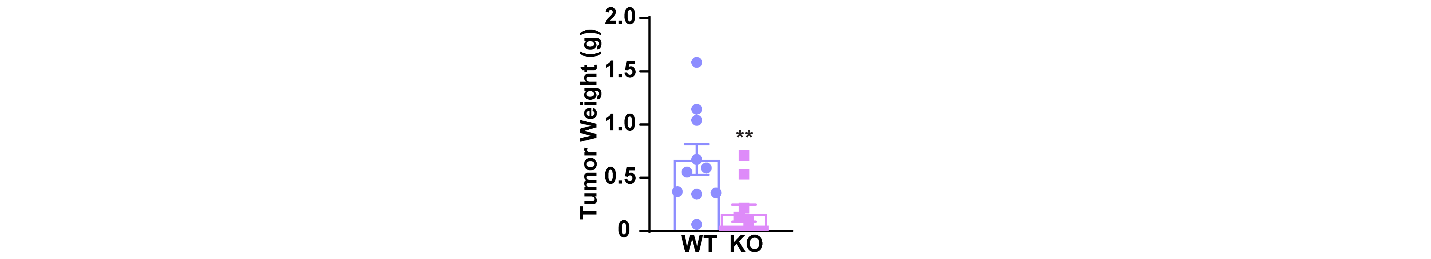


**Figure S1. Tumor weight in WT and KO mice.** Measurement of tumor weight on day 21 post-implantation (n=10 mice per group). Data are presented as mean±SEM. **p<0.01 between the indicated groups based on Student’s t-test.


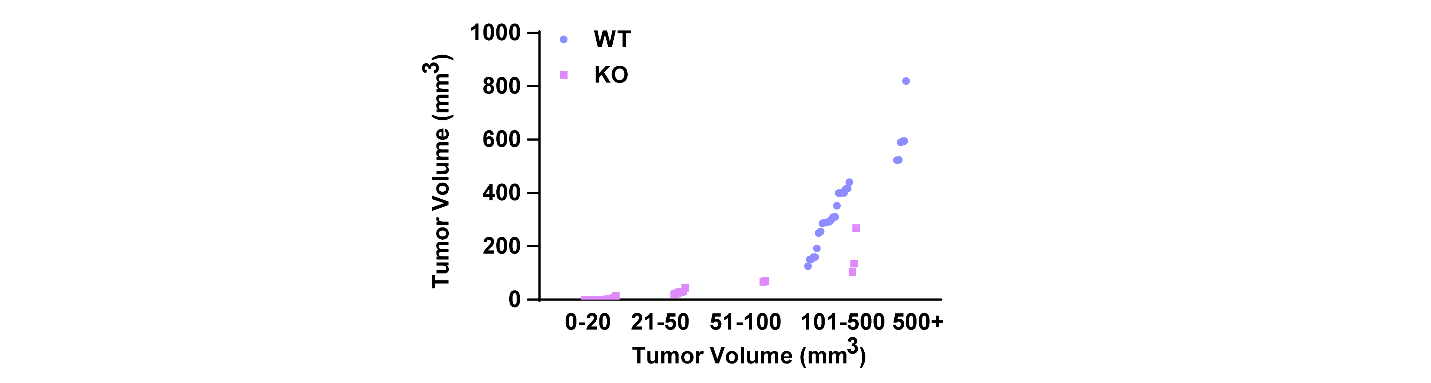


**Figure S2. Tumor size distribution in WT and KO mice.** Measurement of tumor volume on day 21 post-implantation (n=30 mice per group).

**
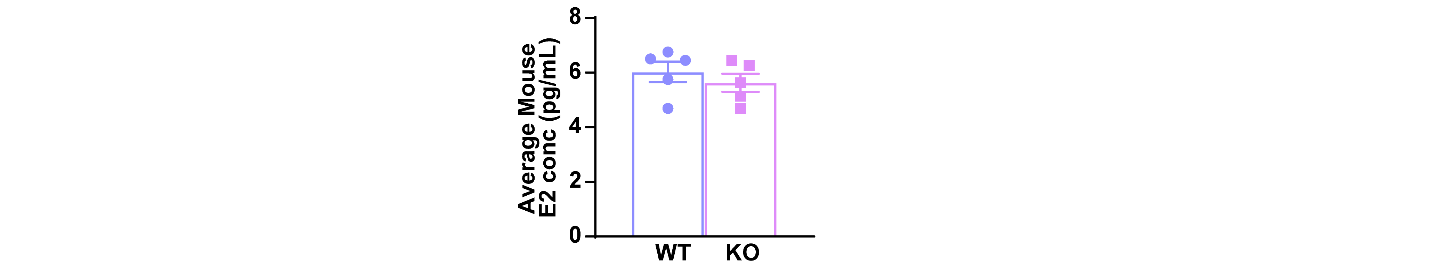
**

**Figure S3. Comparable levels of estradiol in WT and KO mice.** ELISA quantification of plasma circulating estradiol (E2) in WT and EC-AGO1-KO female mice (8-week-old, n=5/group). Data are presented as mean±SEM.


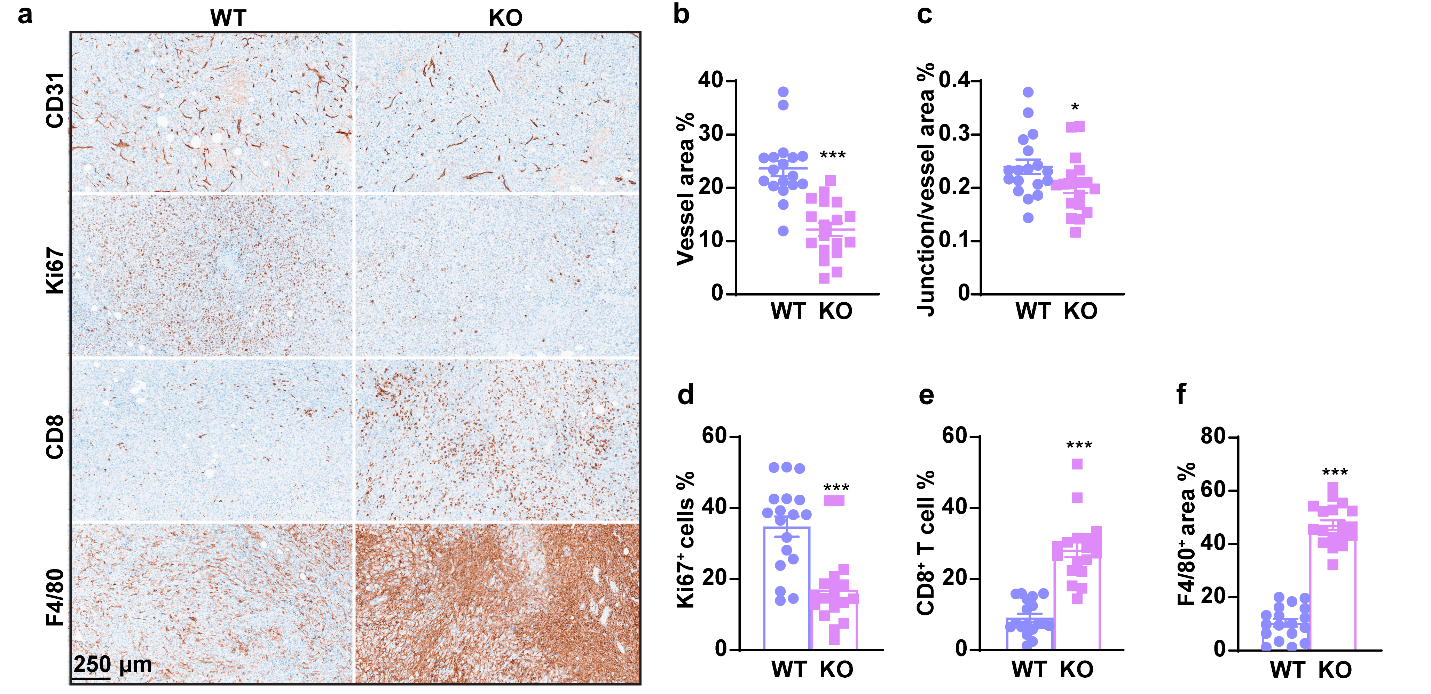


**Figure S4. EC-AGO1 inhibition impairs tumor angiogenesis and modulates the immune microenvironment on day 14**. Female EC-AGO1-KO mice and their WT littermates (16-week-old) were injected with E0771 breast cancer cells, and tumors were harvested at day 14. **a** Representative images of CD31, Ki67, CD8, and F4/80 staining of tumor. **b**, **c** Quantification of vessel percentage and junction/vessel area (18 views from 5-6 tumors/group). **d-f** Quantification of Ki67, CD8 and F4/80-positive area based on 18 views from 5-6 tumors/group). Data are presented as mean±SEM. *p<0.05, ***p<0.001 between the indicated groups based on Student’s t-test (b-f).


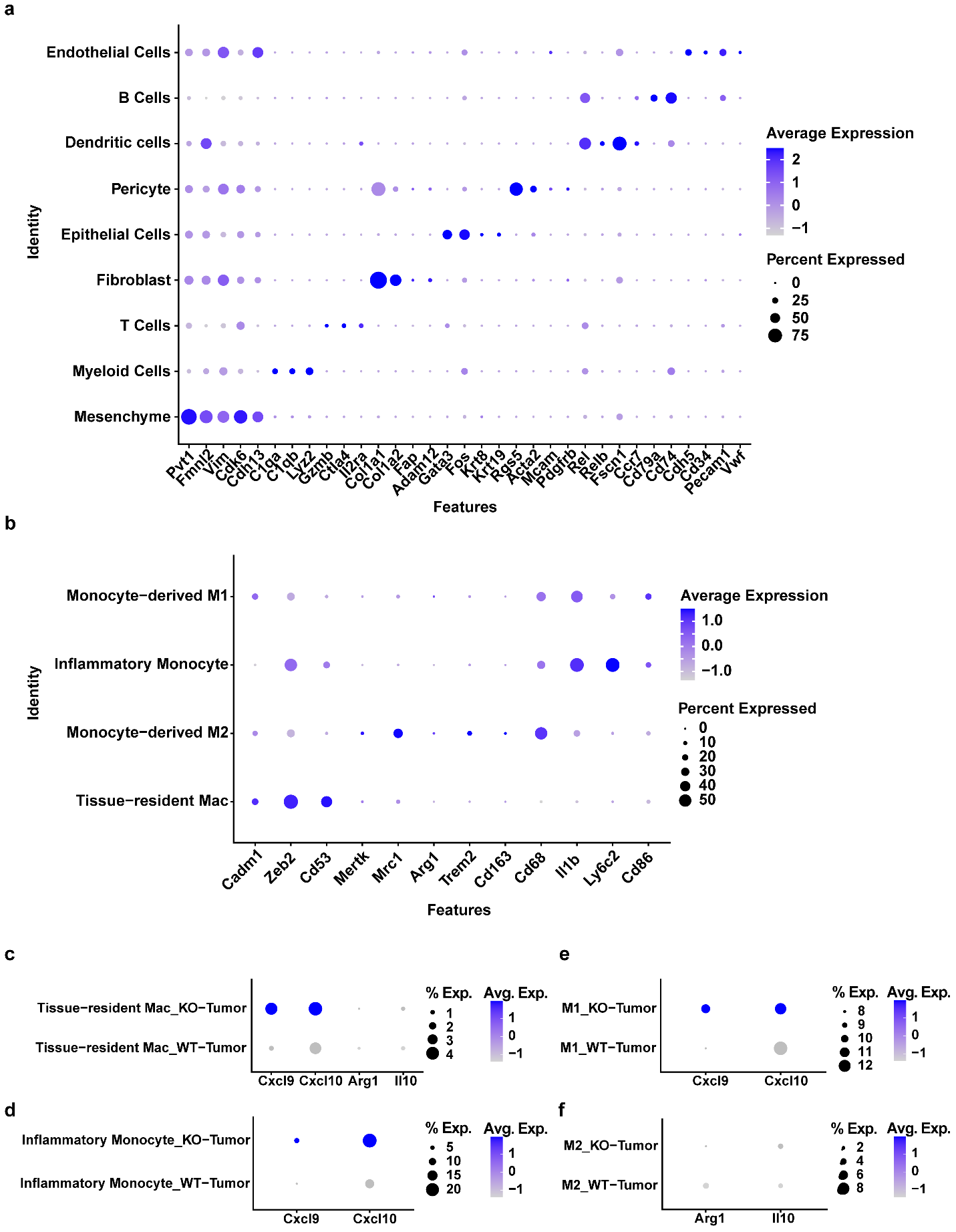


**Figure S5.** **Expression of marker genes for cell annotation in scRNA-seq data.** **a** Dot plot showing expression of cell-marker genes (x-axis) of 9 non-cancer cell clusters within each annotated cell population (y-axis). **b** Dot plot showing expression of cell-marker genes (x-axis) of macrophage subtypes within each annotated cell population (y-axis). **c-f** Dot plots showing pro-inflammatory markers (*Cxcl9/Cxcl10*) and immunosuppressive markers (*Arg1/Il10*) in myeloid subclusters.


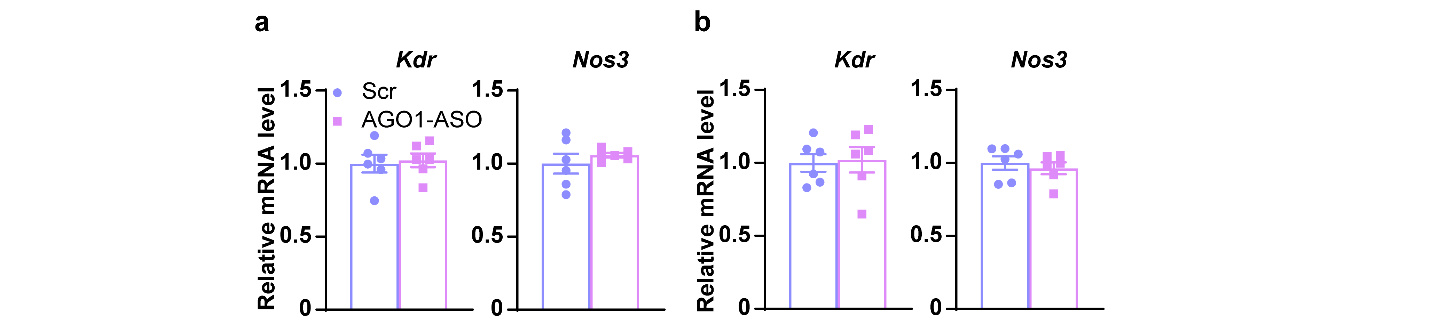


**Figure S6. Effect of EC-AGO1-KD on EC function markers.** MS1 cells were transfected with Scr or AGO1-ASO without (**a**) or with (**b**) co-culture of E0771 cells in Transwell. mRNA levels of *Kdr* and *Nos3* in MS1 cells were quantified by qPCR (n=6). Data are presented as mean±SEM.


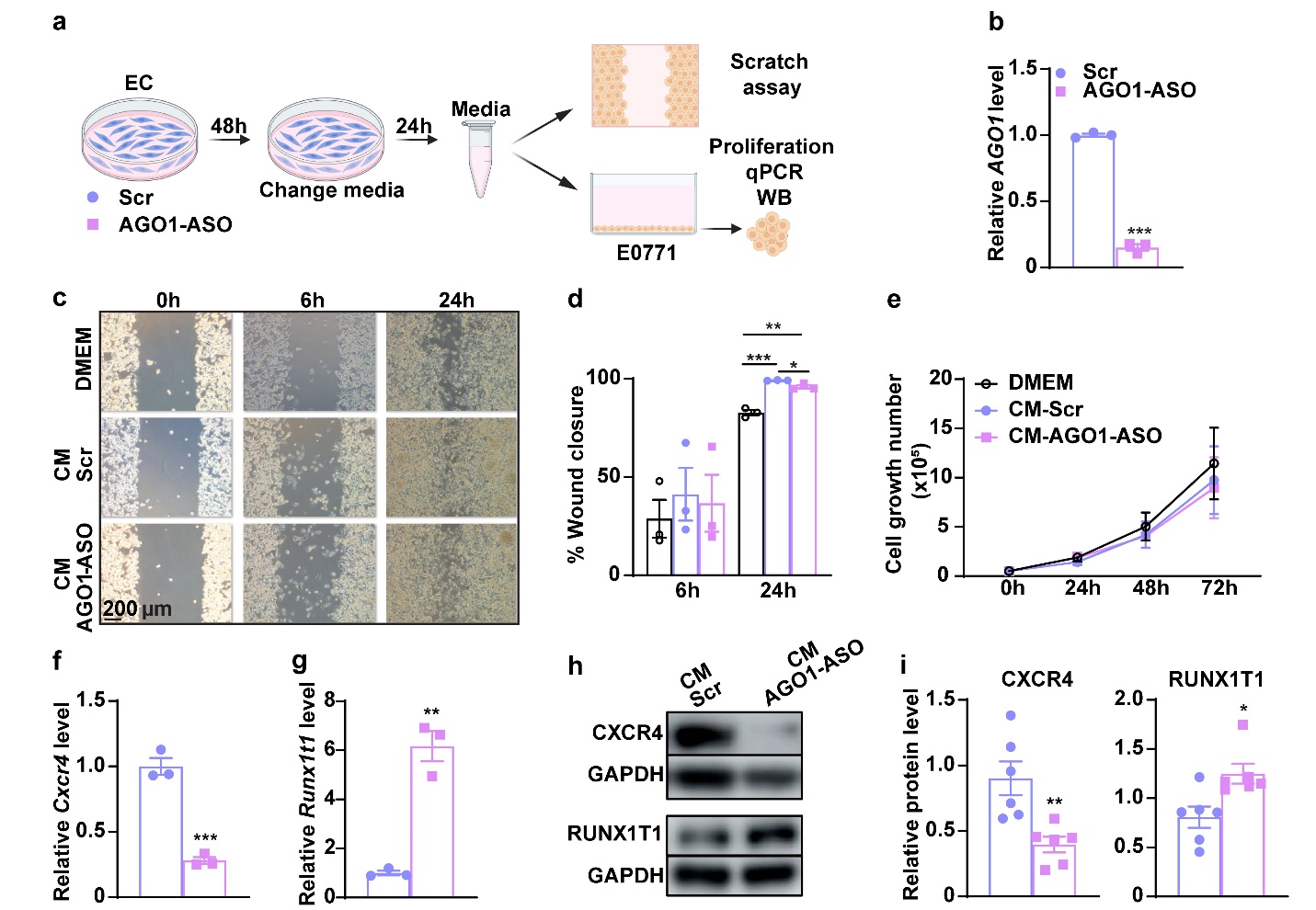


**Figure S7. Effect of conditioned medium from ECs with AGO1-KD on breast cancer cells.** **a** Schematic showing human or mouse ECs (HUVEC or MS1 cells) transfected with control Scr or AGO1- ASO (20 nM) for 48 h, then replaced with fresh media. The conditioned media (CM) were then collected to culture E0771 cells for 24 h. **b** mRNA levels of *AGO1* were quantified by qPCR in HUVECs. **c** Representative images of scratch assay performed with E0771 cells cultured under HUVEC CM at different time points. **d** Quantification of percent (%) wound closure. **e** E0771 cell growth curve cultured with HUVEC CM. **f**, **g** mRNA levels of *Cxcr4* and *Runx1t1* in E0771 were quantified by qPCR cultured under HUVEC CM. **h**, **i** Representative image (**h**) and quantification (**i**) of Western blotting for CXCR4 and RUNX1T1 in E0771 cells cultured with mouse EC CM. Data are presented as mean±SEM (in b, d-g, i). *p<0.05, **p<0.01, ***p<0.001 between the indicated groups based on Student’s t-test (b, f, g, i) or one-way ANOVA (d).

**SUPPLEMENTAL TABLE**

**Table S1. Cell numbers of different cell clusters in tumors from the WT and EC-AGO1-KO mice scRNA-seq data.**

| **Cluster** | **WT** | **KO** |
| --- | --- | --- |
| Mesenchyme Cells | 1588(50.83%) | 1475(38.74%) |
| Myeloid Cells | 718(22.98%) | 1092(26.68%) |
| T Cells | 225(7.20%) | 517(13.58%) |
| Fibroblasts | 238(7.62%) | 172(4.52%) |
| Epithelial Cells | 82(2.62%) | 186(4.89%) |
| Pericytes | 87(2.78%) | 108(2.48%) |
| Dendritic Cells | 81(2.59%) | 96(2.52%) |
| B Cells | 53(1.70%) | 82(2.15%) |
| Endothelial Cells | 52(1.66%) | 79(2.08%) |
| Total | 3807 | 3124 |

**Table S2.** **Sequences of primers used for PCR**

| **Gene** | **Forward** | **Reverse** |
| --- | --- | --- |
| **Human** |  |  |
| *ACTIN* | GCACCACACCTTCTACAATG | ATCACGATGCCAGTGGTAC |
| *AGO1* | CAGGCGGTGAGAAGAAGGAG | ATTGTGAGCTGGCGAATGCT |
| **Mouse** |  |  |
| *Ago1* | GCTGCCCCAGATTCTTCACT | GTCCCTGCTGGGATGTTACC |
| *Cxcr4* | GACTGGCATAGTCGGCAATG | AGAAGGGGAGTGTGATGACAAA |
| *Cxcl9* | GGAGTTCGAGGAACCCTAGTG | GGGATTTGTAGTGGATCGTGC |
| *Cxcl10* | CCAAGTGCTGCCGTCATTTTC | GGCTCGCAGGGATGATTTCAA |
| *Vcam1* | AGTTGGGGATTCGGTTGTTCT | CCCCTCATTCCTTACCACCC |
| *Selp* | GTCTGTCCCGTCACTGGATAC | TCCTCTCTTACCGGGTTACCA |
| *Runx1t1* | ATGCCTGATCGTACCGAGAAG | GTCGTTGGCGTAAATGAGCTG |
| *36B4* | AGATTCGGGATATGCTGTTGGC | TCGGGTCCTAGACCAGTGTTC |
